# Adverse Events Requiring Hospitalization Following Catheter Ablation for Atrial Fibrillation in Heart Failure with versus without Systolic Dysfunction

**DOI:** 10.3390/jcdd11020035

**Published:** 2024-01-23

**Authors:** Naoya Kataoka, Teruhiko Imamura, Takahisa Koi, Keisuke Uchida, Koichiro Kinugawa

**Affiliations:** Second Department of Internal Medicine, University of Toyama, Sugitani, Toyama 930-0194, Japan; nkataoka@med.u-toyama.ac.jp (N.K.); taka1010@med.u-toyama.ac.jp (T.K.); keiuchi1214@yahoo.co.jp (K.U.); kinugawa@med.u-toyama.ac.jp (K.K.)

**Keywords:** heart failure, atrial fibrillation, catheter ablation, major bleeding

## Abstract

Background: The safety and efficacy of atrial fibrillation (AF) ablation in individuals with heart failure (HF) with preserved ejection fraction (EF), particularly concerning the occurrence of post-procedural adverse events necessitating hospitalization, including anticoagulant-associated major bleeding, still lack conclusive determination. Methods: Data from patients with HF and AF who underwent catheter ablation for AF between 2019 and 2022 at our institution were retrospectively reviewed. All participants were divided into an EF < 50% group or an EF ≥ 50% group according to their baseline left ventricular EF. The composite incidence of the clinical events following catheter ablation was compared between the two groups: (1) all-cause death, (2) HF hospitalization, (3) stroke or systemic embolism, and (4) major bleeding. Results: A total of 122 patients (75 years old, 68 male) were included. Of them, 62 (50.8%) patients had an EF ≥ 50%. EF ≥ 50% was an independent predictor of the composite endpoint (adjusted odds ratio 6.07, 95% confidence interval 1.37–26.99, *p* = 0.018). The incidences of each adverse event were not significantly different between the two groups, except for a higher incidence of major bleeding in the EF ≥ 50% group (12.7% vs. 0%, *p* = 0.026). Conclusions: Among patients with HF coupled with AF, the incidence of adverse events following AF ablation proved notably elevated in patients with EF ≥ 50% in contrast to their counterparts with EF < 50%. This disparity primarily stems from a heightened occurrence of major bleeding within the EF ≥ 50% cohort. The strategy to reduce adverse events, especially in patients with EF ≥ 50%, remains the next concern.

## 1. Introduction

Recent accumulating studies have demonstrated that atrial fibrillation (AF) has a significant negative impact on the development of a variety of diseases, including heart failure (HF) and stroke, and increases mortality in patients with a variety of etiologies, regardless of its paroxysmal or persistent classification [1]. In particular, AF has a strong association with HF. AF causes new-onset HF in 30% of patients with AF and vice versa [2].

The association between AF and HF appears to be influenced by the level of left ventricular ejection fraction (EF). The prevalence of AF is higher in patients with preserved EF [3]. The impact of AF on cardiovascular mortality is greater in patients with preserved EF than in those with reduced EF [4]. However, a detailed mechanism to explain such a different impact of AF between patients with preserved EF and those with reduced EF remains uncertain.

Recently, catheter ablation for AF has been aggressively pursued in patients with HF with reduced EF because of its accumulating positive evidence: AF ablation facilitates cardiac reverse remodeling and improves morbidity and mortality in this cohort. On the contrary, the clinical implications of AF ablation in patients with HF with preserved EF remain controversial [5,6]. The large-scale study using nationwide healthcare data in the US demonstrated that early mortality after AF ablation was comparable between patients with preserved EF and those with reduced EF [7]. Other studies reported a controversial prognostic impact of AF ablation, regardless of the EF category [8,9].

One of the debates is the definition of the study endpoint. Most previous studies defined the primary endpoint as all-cause death, cardiovascular death, HF recurrence, stroke, or a composite of these [3,4,5,6,7,10]. Major bleeding is one of the most important clinical outcomes in patients with AF, especially gastrointestinal events, including bleeding, which is the second cause of 30-day readmission after AF ablation in patients with HF [11]. However, most of the previous studies rarely evaluated this endpoint. Given the different background physiology, we hypothesized that the prognostic impact of AF ablation may differ between those with preserved EF and those with reduced EF, especially in the incidence of major bleeding.

Therefore, the present study aimed to investigate the prognostic impact of AF ablation in HF patients with preserved EF compared with those without preserved EF and attempted to discuss the clinical implications of AF ablation in HF patients with preserved EF.

## 2. Methods

### 2.1. Study Population

Consecutive 290 patients who received AF ablation between November 2019 and December 2022 at our institution were considered to be included in this retrospective cohort study. Patients who met the following criteria were finally enrolled: (1) patients with HF, which was diagnosed according to the Framingham criteria, including HF-related symptoms such as leg edema, dyspnea of effort, and nocturnal orthopnea [12]; (2) patients with non-valvular AF at baseline, which was detected in the surface twelve-lead electrocardiograms within one year.

The exclusion criteria were defined as follows: (1) patients without HF who did not satisfy the Framingham criteria due to the lack of HF symptoms and/or signs, irrespective of the plasma levels of B-type natriuretic peptide; (2) patients who had received other cardiovascular intervention within three months; (3) patients with a history of catheter ablation for AF. The present study was approved by the institutional review board at the University of Toyama. Informed consents were waived given their retrospective nature without any intended interventions or opt-out methods.

### 2.2. Baseline Characteristics and Classification Based on Left Ventricular EF

Clinical characteristics, including demographics, laboratory, and echocardiographic data, were retrieved from the electronic medical record. Variables obtained by an echocardiogram were collected within three months before the procedure. 

The enrolled subjects were classified into the following two groups according to their left ventricular EF levels: the EF ≥ 50% group and the EF < 50% group.

### 2.3. Ablation Procedures

The patient underwent catheter ablation while maintaining the administration of warfarin. On the day of the procedure, whether the patient was on a once-daily or twice-daily regimen of direct oral anticoagulants, the medication was temporarily withheld only in the morning. The transseptal puncture was adopted for the left atrial approach, and heparin was infused intravenously with a target-activated clotting time > 300 s. Following the obtain of three-dimensional geometry utilized by the Advisor HD Grid Mapping Catheter (Abbott Inc., Abbott Park, IL, USA) or PENTARAY NAV ECO High-Density Mapping Catheter (Biosense Webster Inc., Diamond Bar, CA, USA), pulmonary vein isolation underwent using the TactiCath Contact Force Ablation Catheter, Sensor Enabled (Abbott Inc.), or ThermoCool SmartTouch SF Catheter (Biosense Webster Inc.). Additional linear ablation was determined by each operator. In cases of cryoballoons utilized by the Arctic Front Advance (Medtronic Inc., Minneapolis, MN, USA), each pulmonary vein was isolated individually, and the roof line using the balloon was added if the operators determined it was necessary. The Perclose ProGlide suture-mediated closure system (Abbott Inc.) was utilized for the femoral venous access site closure in all patients.

### 2.4. Study Outcomes

The primary endpoint was defined as the composite of all-cause death, unplanned HF hospitalization, stroke or systemic embolism, and major bleeding. The major bleeding event was defined as intracranial hemorrhage, major gastrointestinal bleeding, or bleeding from other sites that required blood transfusion. Day 0 was defined at the time of index discharge. All patients were followed at our institute or affiliated institutions from the index discharge until February 2023.

Each clinical event of the primary endpoint was counted individually and defined as the secondary endpoint, in addition to the occurrence of any atrial tachyarrhythmias. Atrial tachyarrhythmias were counted from the end of the post-procedural 90-day blanking period. Twelve-lead surface electrocardiograms, 24-hour or two-week Holter monitors, or portable electrocardiograms (OMRON Corp., Kyoto, Japan) were utilized for detecting atrial tachyarrhythmias. Echocardiographic parameters were rerecorded three months following the procedure.

### 2.5. Statistical Analysis

Continuous data were described as the median with the interquartile range and were compared using the Mann-Whitney’s test for non-normally distributed variables. In cases where the data were normally distributed, the unpaired t-test was employed. Categorical data were expressed as numbers and percentages and were compared using the Chi-squared test.

The cumulative incidence of clinical outcomes was stratified according to the EF levels (EF ≥ 50% vs. EF < 50%) and compared between the two groups using a log-rank test. Cox proportional hazard ratio regression analyses were performed to evaluate the prognostic impact of EF ≥ 50% following AF catheter ablation, which was adjusted for other potential confounders according to the previous literature [6,7,10,13]. A two-sided P-value < 0.05 was considered statistically significant. Data analyses were performed using JMP ver. 14 (SAS, Cary, NC, USA).

## 3. Results

### 3.1. Patient Characteristics 

Among the 290 patients screened, 122 subjects finally met the inclusion criteria. These patients underwent ablation procedures conducted by three doctors (N.K., T.K., and K.U.), all of whom were well-trained physicians. The median age was 75 (70, 79) years old, and 54 (44%) were female (Table 1). All patients had HF with a serum N-terminal pro-B-type natriuretic peptide level of 1403 (570, 3060) pg/mL. All patients had AF, including 48 (39%) paroxysmal types. The median CHADS2 score was 3 (2, 3), and the CHA2DS2-VASc score was 4 (3, 5).

Of these, 62 (50.8%) patients were assigned to the EF ≥ 50% group (Table 1). Patients in the EF ≥ 50% group had a higher age, a higher prevalence of hypertension, and a lower prevalence of cardiomyopathy. The prevalence of anti-HF medication was lower than that of those with EF < 50%. The serum N-terminal pro-B-type natriuretic peptide level was lower in the EF ≥ 50% group. The estimated stroke/bleeding risk, calculated by the CHADS2 score, CHA2DS2-VASc score, and HAS-BLED score, were not significantly different between the EF ≥50% group and the EF < 50% group (*p* > 0.05 for all). The incidence of antiplatelet therapy was similar between those with EF ≥ 50% and EF < 50%.

### 3.2. Catheter Ablation for AF

The procedure of catheter ablation is detailed in Table 2. Pulmonary vein isolation was completed in all patients. The prevalence of cryoballoon ablation was not significantly different between the patients with EF ≥ 50% and those with EF < 50% (*p* = 0.892). Additional linear ablation was less frequently performed in patients with EF ≥ 50% (*p* = 0.002). In the subjects of this study, there were no occurrences of acute complications related to the procedure, such as pericardial effusion, cardiac tamponade, symptomatic cerebrovascular thromboembolisms, or left atrium-esophageal fistula.

### 3.3. Primary Endpoint

**Figure 1:**
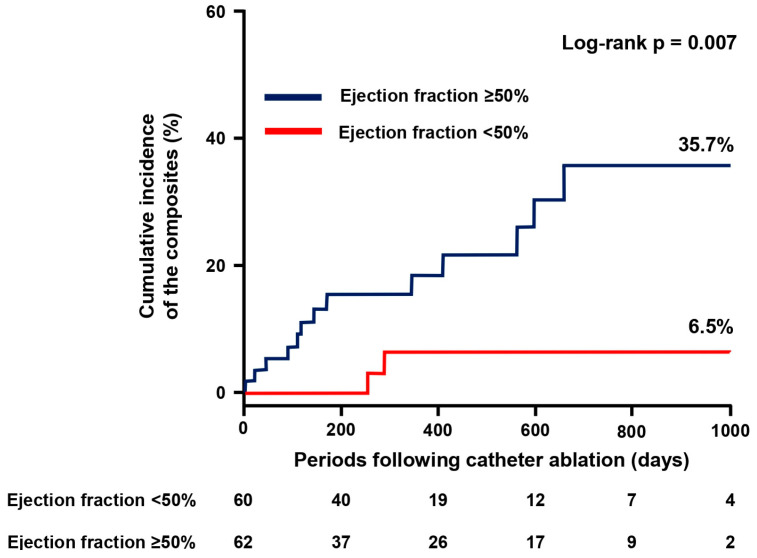
Cumulative incidence of the primary endpoint. The primary composite endpoint consists of all-cause death, heart failure hospitalization, stroke or systemic embolism, and major bleeding.

Following the index discharge after catheter ablation, patients were followed for 305 (143–587) days on average. During the observation period, 15 patients achieved the composite primary endpoint and encountered either of the following adverse events, consisting of three all-cause deaths, ten HF hospitalizations, two strokes/systemic embolisms, or four major bleedings. Of them, four patients had multiple events at once. The cumulative incidence of the primary composite endpoint was significantly higher in the EF ≥ 50% group compared with the EF < 50% group (35.7% vs. 6.5%, *p* = 0.007; Figure 1).

EF ≥ 50% was associated with the occurrence of a primary endpoint with an odds ratio of 6.07 (95% confidence interval 1.37–26.99, *p* = 0.018). The prognostic impact of EF ≥ 50% remained following adjustment for each potential confounder (*p* < 0.05; Table 3).

### 3.4. Secondary Endpoint

**Figure 2:**
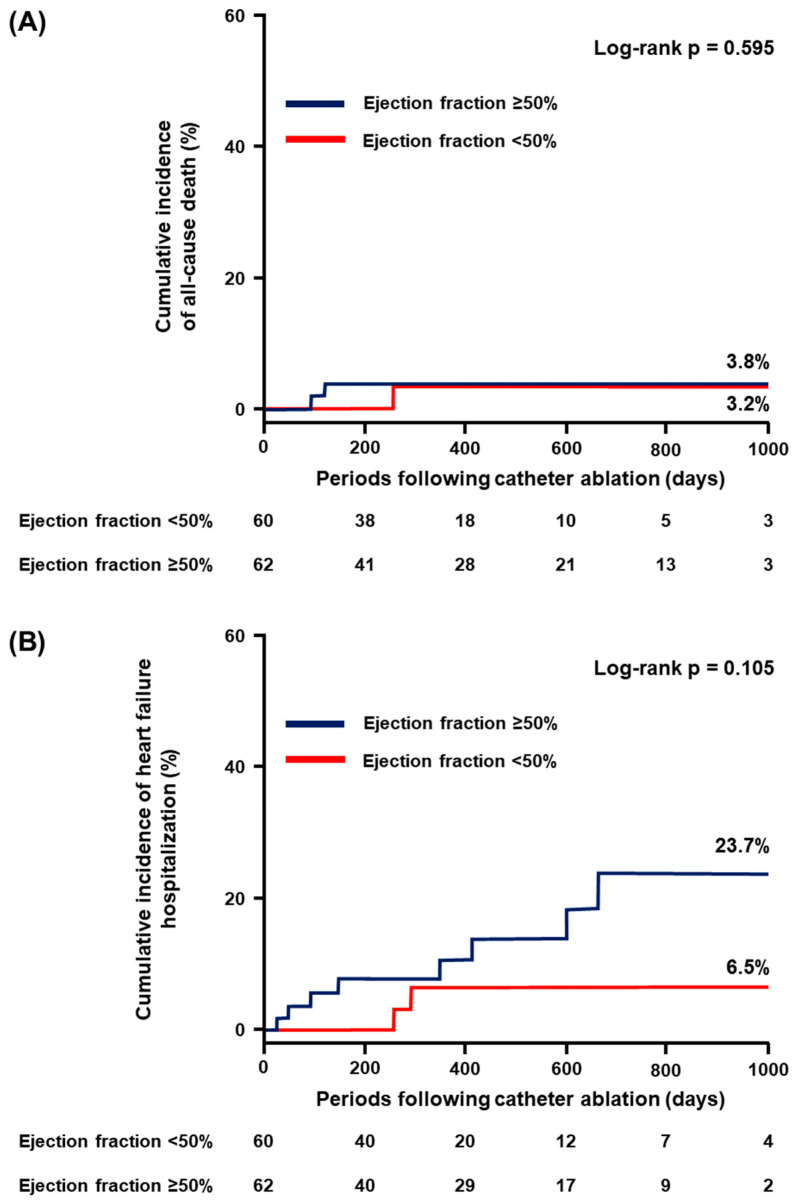

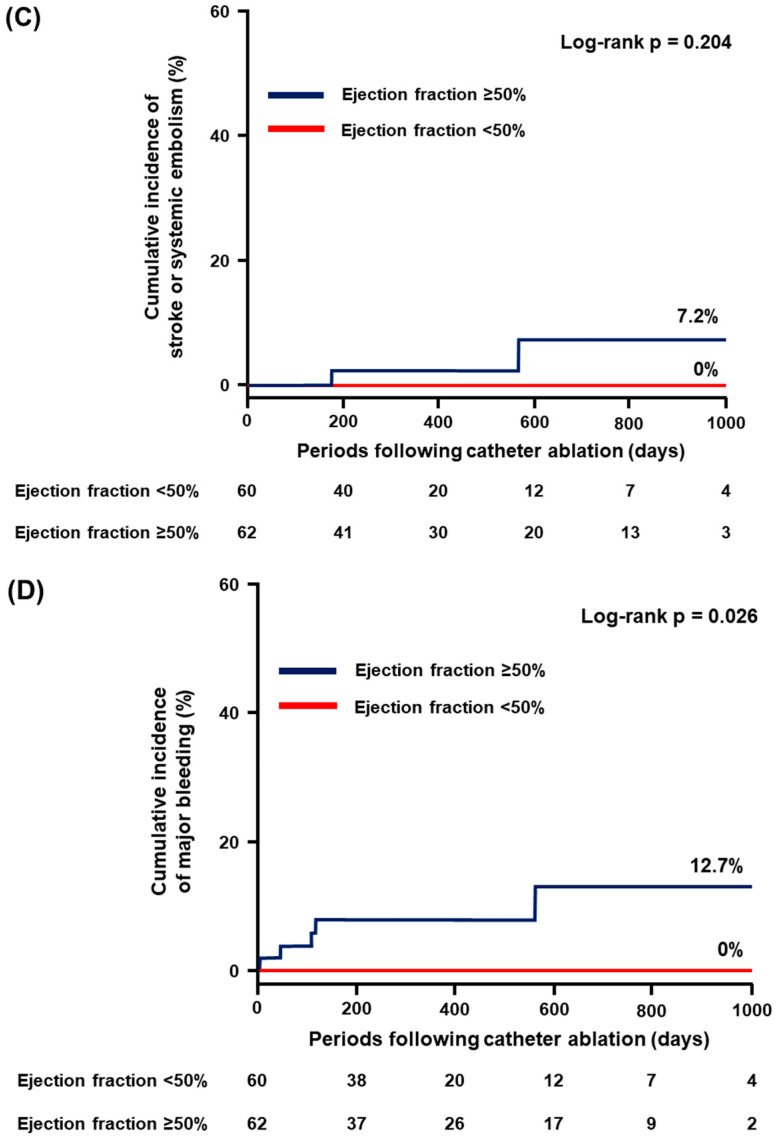
Cumulative incidence of the secondary endpoint: (**A**) all-cause death; (**B**) heart failure hospitalization; (**C**) stroke or systemic embolism; and (**D**) major bleeding.

EF ≥ 50% did not significantly stratify the individual incidence of all-cause death, HF hospitalization, and stroke/systemic embolism (*p* > 0.05 for all; Figure 2A–C). On the contrary, the cumulative incidence of major bleeding was significantly higher in patients with EF ≥ 50% compared to those with EF < 50% (12.7% vs. 0%, *p* = 0.026; Figure 2D).

**Figure 3:**
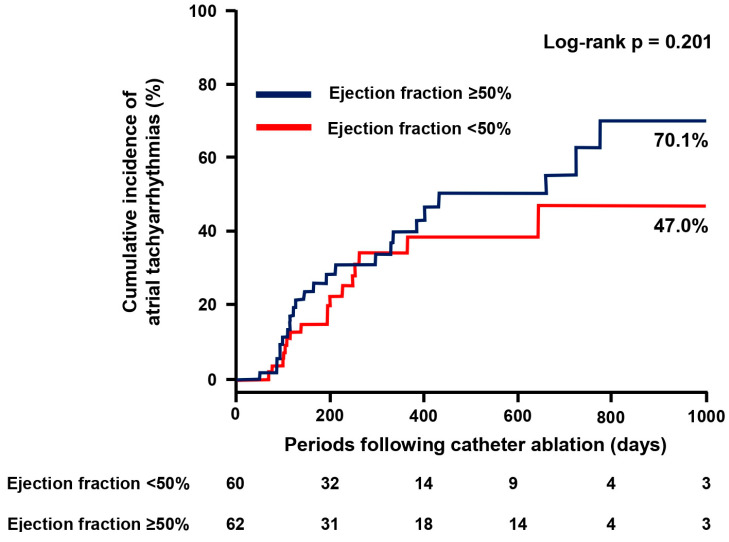
Cumulative incidence of atrial tachyarrhythmias.

The cumulative incidence of the recurrence of atrial tachyarrhythmias was not significantly different between those with EF ≥ 50% and those with EF < 50% (*p* = 0.201; Figure 3). Among them, no patients initiated new anti-arrhythmic drugs. The degree of increase in the left ventricular EF was significantly lower in the EF ≥ 50% group compared with the EF < 50% group (−1 [6–7]% vs. 19 [6–26]%, *p* < 0.001).

The detailed cases of the primary endpoint occurrence are displayed in Table 4. The most frequent event was HF worsening along with AF recurrence in six (40%) patients. The second was gastrointestinal bleeding in four (27%) patients, all of whom were classified in the EF ≥ 50% (0% in the EF < 50% vs. 6% in the EF ≥ 50%, *p* = 0.045). Of them, two (50%) patients required hospitalization due to HF worsening owing to anemia related to gastrointestinal bleeding. Furthermore, there were no cases of major bleeding in the EF < 50%.

## 4. Discussion

We investigated the clinical outcomes following catheter ablation for AF in patients with preserved EF by comparing them to those with HF and EF < 50%. Patients with preserved LEF were older, had a higher prevalence of hypertension, and received anti-HF medications less frequently as compared to those with EF < 50%. Pulmonary vein isolation was performed successfully in all patients. The additional linear ablation was less frequently performed in patients with EF ≥ 50%. The incidence of the primary composite endpoint was higher in patients with preserved EF. The breakdowns of each endpoint were not significantly different in their incidence between those with EF ≥ 50% and those with EF < 50%, except for a higher incidence of major bleeding in the EF ≥ 50% group. The incidence of post-ablation atrial tachyarrhythmias was not significantly stratified by the EF levels.

### 4.1. Impacts of AF Ablation on Improving EF

Current guidelines recommend catheter ablation for AF in the setting of systolic HF as class IIb or over levels [14,15]. Many studies consistently demonstrated favorable clinical outcomes following AF ablation in patients with systolic HF, accompanied by improvement in left ventricular EF [16]. Left ventricular EF is an established predictor of mortality and morbidity in patients with systolic HF. Improvement in left ventricular EF is associated with a future mortality reduction [17,18].

We consistently observed a 19% increase in left ventricular EF following AF ablation in patients with EF < 50%. Such reverse remodeling, dominantly due to recovery and maintenance of sinus rhythm, should be one of the major reasons for favorable clinical outcomes following AF ablation in this cohort. On the contrary, hemodynamic improvement via an increase in EF is hardly expected in patients with preserved EF. Thus, the detailed impact of AF ablation in patients with HF and preserved EF remains under debate.

### 4.2. Maintenance of Sinus Rhythm Following AF Ablation

Maintenance of sinus rhythm following AF ablation is another concern when discussing the implications of AF ablation. Consistently with other previous studies, the incidence of re-current atrial tachyarrhythmias was not significantly different between those with EF ≥ 50% and those with EF < 50% [7,10]. The linear or intra-cardiac potential-guided ablation in addition to pulmonary vein isolation, which was more frequently performed in patients with EF < 50% in this study, might not necessarily reduce the incidence of AF recurrence [19].

### 4.3. How to Assess the Impact of AF Ablation on HF and Preserved EF

One of the challenges to correctly assessing the impact of AF ablation in patients with preserved EF is the accurate definition of HF. Sometimes the signs and symptoms of AF mimic those of HF. It is sometimes challenging to appropriately distinguish HF + AF from AF alone, particularly when patients’ EF is preserved. Thus, many large-scale studies might have included those with AF alone and assumed them to be HF cohorts [12]. In this study, we adopted strict inclusion/exclusion criteria to exclude patients with AF alone. For example, patients with elevated natriuretic peptide were excluded when they did not have any HF symptoms or signs. Moreover, HF with preserved EF includes heterogeneous groups that may confound clinical outcomes [20].

Another challenge to assessing the impact of AF ablation in patients with HF with preserved EF is the definition of the endpoint. The incidence of all-cause death, HF recurrence, and stroke/systemic embolism following AF ablation was superior in patients with pre-served EF to those with reduced EF in several large registry studies [5,6,8,9,10,18]. The US national database study demonstrated a similar incidence of all-cause death and HF recurrence following AF ablation between those with preserved EF and those with reduced EF [7].

However, gastrointestinal bleeding is one of the most important adverse events requiring hospitalization in AF patients who have undergone catheter ablation [11]. Therefore, we added major bleeding as one of the main clinical endpoints in this study, and the incidence of major bleeding was higher in patients with preserved EF.

### 4.4. Clinical Outcomes Following Catheter Ablation

Some guidelines propose catheter ablation for AF in patients with HF, irrespective of EF [21]. However, our results directed a question at the recommendation in patients with preserved EF due to the unsatisfactory outcomes compared with those in reduced EF patients. Although several favorable effects of ablation for patients with preserved EF were proven, further ingenuities are warranted to be close to the outcomes in patients with reduced EF [8,9].

In this study, the most frequent cause of reaching the endpoints was AF-recurrence-related HF. Although ablation-related technical ingenuities to reduce atrial tachyarrhythmia recurrence should be needed, the efficacies of additional ablation apart from pulmonary vein isolation seem to be limited, as described above. Therefore, the time to explore other approaches to reduce AF recurrence in patients with HF is coming.

The second reason for reaching the endpoints was gastrointestinal bleeding, especially in the HF with EF ≥ 50% group. A previous paper reported that gastrointestinal events were the second major cause of re-hospitalization following AF ablation in HF patients [11]. Medi-care data targeted patients newly diagnosed with AF, and demonstrated that bleeding occurred comparably irrespective of EF in patients with and without HF [22]. In this study, the incidence of major bleeding was higher in the HF with EF ≥ 50% group than in the HF with EF < 50% group, despite the administration rates of anticoagulants and HAS-BLED score being comparable between the two.

### 4.5. Post-Ablation Gastrointestinal Bleeding

One of the possible explanations for the high incidence of major bleeding in preserved EF patients, despite similar HAS-BLED scores between the EF ≥ 50% and EF < 50% groups, is several differences in baseline characteristics. The risk factors associated with anticoagulant-related bleeding encompass advanced age, low body weight, renal dysfunction, diabetes mellitus, and concurrent use of aspirin or NSAIDs [23]. Although HF with EF ≥ 50% remained an independent predictor of the endpoints even after adjusting for several cofounders, a high age and a high incidence of hypertension in the EF ≥ 50% group should have affected the incremental risk of major bleeding in this cohort [22]. Since there were no patients who initiated new antiarrhythmic drugs after ablation, it is presumed that there was no increased risk of bleeding due to interactions between direct oral anticoagulants and antiarrhythmic drugs.

Another possible reason is an elevation of venous pressure leading to gastrointestinal congestion. Although we did not assess hemodynamic pressure data, right atrial pressure elevation could predict gastrointestinal bleeding in patients with preserved EF and have additional predictive power on HAS-BLED [24]. A more accurate risk calculation for predicting bleeding alternatives to HAS-BLED should be investigated in future studies [25].

Moreover, the inherent distinctions in the progression of non-cardiovascular events between HF with reduced EF and with preserved EF could potentially influence the out-comes of this study. More than 80% of patients with reduced EF ultimately experience cardiovascular death, whereas a considerable number of patients with HF and preserved EF succumb to death resulting from intracranial bleeding and multisystem organ failure [26]. These previously reported data may support our results that major bleeding was higher in patients with EF ≥ 50% compared with those with EF < 50%.

Inflammation or extracellular matrix remodeling might also contribute to the difference in bleeding. Chronic inflammation plays a crucial role in the pathogenesis of heart failure with preserved EF [27]. Furthermore, the persistence of inflammation leads to extracellular matrix remodeling [28]. Both inflammation and extracellular matrix degeneration are known contributors to bleeding [29].

### 4.6. Clinical Perspective

The findings suggest that the mitigation of major bleeding events, particularly in heart failure patients with an EF ≥ 50%, may play a crucial role in improving prognosis. From recent data, the procedure-related complications of catheter ablation for AF have shown a decreasing trend [30]. Specifically, the use of vascular ultrasound for punctures should contribute to reducing complications related to the vascular access site [31,32,33]. Despite the implementation of echo-guided puncture in this study, a notable tendency towards bleeding events, particularly gastrointestinal bleeding, was observed in EF ≥ 50% subjects. The left atrial appendage occlusion is the established method for reducing major bleeding in patients receiving anticoagulant therapy [34]. Although approximately 10% of the enrolled subjects underwent left atrial appendage occlusions, the cases that demonstrated gastrointestinal bleeding following catheter ablation did not receive that therapy at the time of the event occurrence. Proactive adaptation, particularly in patients with preserved EF, may contribute to the reduction of major bleeding following AF ablation in HF patients. Alternatively, abelacimab, a novel anticoagulant as a factor XIa inhibitor, has the potential to mitigate bleeding events in atrial fibrillation patients undergoing anticoagulant therapy, offering an alternative to commercially available anticoagulants [35].

### 4.7. Study Limitations

Several limitations were raised in this study. First, this was a single-center and retrospective study, leading to a selection bias for the procedure that might affect clinical outcomes. Second, there were several variables that differed between the two groups; however, with only 15 (12%) events occurring for the endpoints, this resulted in low statistical power during the multivariable analysis. As a consequence, we cannot rule out the existence of any other unconsidered potential confounders. Third, distinguishing symptoms related to HF from those attributable to AF is challenging during the AF rhythm. Therefore, some cases in this study may have been misdiagnosed as HF. Fourth, owing to the enrollment of the cases before the approval of sodium-glucose cotransporter 2 inhibitors use for HF in Japan, the relatively low administration rate of that in this study. The facts might affect the AF recurrence rate because sodium-glucose cotransporter 2 inhibitors could reduce AF recurrence [36]. Finally, the mechanisms underlying these differences between HF with EF ≥ 50% and EF < 50% remain unclear. The advanced age and higher incidence of hypertension in patients with EF ≥ 50% in this cohort, in contrast to those with EF < 50%, may potentially contribute to vascular fragility, thereby leading to bleeding.

## 5. Conclusions

Patients with HF exhibiting preserved EF do not experience comparable beneficial outcomes following catheter ablation of AF when juxtaposed with individuals manifesting systolic dysfunction, notably regarding occurrences of major bleeding. It becomes evident that supplementary interventions beyond medication and AF ablation are necessary to enhance clinical outcomes within this specific patient cohort.

## Figures and Tables

**Table 1:** Baseline clinical characteristics.

Variable	Overall	EF < 50%	EF ≥ 50%	*p* Value
Number of patients	122	60	62	
Age (years)	75 [70–79]	74 [64–79]	77 [72–80]	0.020
Female, n (%)	54 (44)	25 (42)	29 (47)	0.570
Body mass index (kg/m^2^)	22.2 [19.6–25.4]	21.3 [19.1–24.2]	23.7 [20.2–25.8]	0.050
Paroxysmal AF, n (%)	48 (39)	19 (32)	29 (47)	0.088
CHADS2	3 [2–3]	3 [2–3]	3 [2–3]	0.674
CHA2DS2-VASc	4 [3–5]	4 [3–5]	4 [3–5]	0.089
HAS-BLED	1 [1–2]	1 [1–2]	1 [1–2]	0.521
Daily drinking, n (%)	38 (31)	18 (30)	20 (32)	0.788
Daily smoking, n (%)	20 (16)	10 (17)	10 (16)	0.936
Comorbidities				
Hypertension, n (%)	69 (57)	26 (43)	43 (69)	0.004
Diabetes mellitus, n (%)	36 (30)	18 (30)	18 (29)	0.907
Dyslipidemia, n (%)	39 (32)	18 (30)	21 (34)	0.647
Ischemic etiology, n (%)	22 (18)	11 (18)	11 (18)	0.932
Primary cardiomyopathies, n (%)	32 (26)	27 (45)	5 (8)	<0.001
Valvular heart diseases, n (%)	14 (11)	4 (7)	10 (16)	0.101
CIEDs, n (%)	19 (16)	15 (25)	4 (6)	0.005
Pacemaker, n (%)	7 (6)	5 (8)	2 (3)	0.225
Implantable cardioverter defibrillator, n (%)	10 (8)	8 (13)	2 (3)	0.042
Cardiac resynchronization therapy, n (%)	10 (8)	10 (17)	0 (0)	0.001
Left atrial appendage closure devices, n (%)	14 (11)	7 (12)	7 (11)	0.948
Medications				
ACEi or ARB, n (%)	63 (52)	30 (50)	33 (53)	0.722
ARNI, n (%)	28 (23)	20 (33)	8 (13)	0.007
β-blockers, n (%)	103 (84)	57 (95)	46 (74)	0.002
MRA, n (%)	59 (48)	38 (63)	21 (34)	0.001
SGLT2 inhibitor, n (%)	52 (43)	34 (57)	18 (29)	0.002
Loop diuretics, n (%)	76 (62)	38 (63)	38 (62)	0.906
Anti-arrhythmic drugs				
Amiodarone, n (%)	36 (30)	22 (37)	14 (23)	0.088
Bepridil, n (%)	5 (4)	1 (2)	4 (6)	0.183
Sodium channel blockers, n (%)	2 (2)	0 (0)	2 (3)	0.161
Anticoagulant				
Dabigatran, n (%)	32 (26)	15 (25)	17 (27)	0.761
Apixaban, n (%)	43 (35)	23 (38)	20 (32)	0.483
Edoxaban, n (%)	30 (25)	15 (25)	15 (24)	0.918
Rivaroxaban, n (%)	5 (4)	1 (2)	4 (6)	0.183
Warfarin, n (%)	12 (10)	6 (10)	6 (10)	0.952
Antiplatelet, n (%)	25 (20)	12 (20)	13 (21)	0.895
Echocardiographic parameters				
LVEF (%)	50 [37–64]	37 [29–44]	64 [55–68]	<0.001
LV end diastolic diameter (mm)	49 [45–54]	53 [49–60]	46 [42–49]	<0.001
LV end systolic diameter (mm)	36 [29–43]	42 [37–50]	29 [26–33]	<0.001
Left atrial diameter (mm)	45 [40–50]	45 [39–50]	45 [40–50]	0.852
Laboratory data				
Aspartate aminotransferase, mg/dL	24 [20–30]	23 [18–33]	24 [20–28]	0.743
Alanine aminotransferase, mg/dl	18 [12–23]	19 [13–26]	17 [12–23]	0.192
γ-Glutamyl transpeptidase, mg/dL	30 [22–56]	32 [23–55]	29 [19–58]	0.313
Creatinine, mg/dL	1.0 [0.8–1.3]	1.0 [0.9–1.4]	0.9 [0.8–1.3]	0.055
Estimated GFR, mL/min/1.73 m^2^	51 [38–63]	48 [36–63]	52 [42–63]	0.281
Sodium, mEq/L	140 [138–142]	139 [137–142]	141 [140–142]	0.004
Potassium, mEq/L	4.4 [4.1–4.6]	4.4 [4.1–4.6]	4.4 [4.1–4.6]	0.655
Hemoglobin, g/dL	13.1 [11.6–14.3]	13.5 [11.8–14.7]	12.7 [11.1–14.0]	0.059
N-terminal pro-B-type natriuretic peptide, pg/mL	1403 [570–3060]	2242 [651–3887]	1188 [469–1896]	0.011
B-type natriuretic peptide, pg/mL	184 [85–413]	223 [86–476]	169 [85–348]	0.215
Troponin I, pg/mL	10.0 [0–26.8]	11 [0–26]	10 [0–28]	0.763
Uric acid, mg/dL	6.1 [4.8–7.2]	5.8 [4.7–6.9]	6.2 [4.9–7.5]	0.253
C-reactive protein, mg/dL	0.10 [0.05–0.29]	0.09 [0.06–0.64]	0.11 [0.04–0.23]	0.191

ACE, angiotensin-converting enzyme inhibitor; AF, atrial fibrillation; ARB, angiotensin receptor blocker; CIEDs, cardiac implantable electronic devices; EF, ejection fraction; GFR, glomerular filtration rate; HF, heart failure; LV, left ventricular; MRA, mineralocorticoid receptor antagonists; SGLT2, sodium-glucose cotransporter 2.

**Table 2:** Procedural details.

	Overall	EF < 50%	EF ≥ 50%	*p* Value
Cryoballoon ablation, n (%)	78 (64)	38 (63)	40 (65)	0.892
Pulmonary vein isolation, n (%)	122 (100)	60 (100)	62 (100)	
Additional linear ablation, n (%)	68 (56)	42 (70)	26 (42)	0.002
Roofline with cryoballoon, n (%)	28 (23)	20 (33)	8 (13)	0.007
Posterior wall isolation, n (%)	28 (23)	15 (25)	13 (21)	0.597
Mitral isthmus block line, n (%)	1 (0)	1 (2)	0	0.307
Superior vena cava isolation, n (%)	1 (0)	1 (2)	0	0.307
Cavotricuspid isthmus block line, n (%)	15 (12)	8 (13)	7 (11)	0.731

**Table 3:** Multivariate analysis to predict the composite endpoint.

	Hazard Ratio	95% CI	*p* Value
Model 1			
HF with EF ≥ 50%	6.18	1.36–28.04	0.018
CHA2SD2-VASc Score (1 point increase)	0.91	0.63–1.32	0.622
Model 2			
HF with EF ≥ 50%	6.53	1.45–29.28	0.014
HAS-BELD Score (1 point increase)	1.63	0.91–2.70	0.073
Model 3			
HF with EF ≥ 50%	9.27	1.82–47.18	0.007
Primary cardiomyopathies	2.41	0.69–8.40	0.169
Model 4			
HF with EF ≥ 50%	7.23	1.40–37.30	0.018
CIEDs	1.59	0.30–8.23	0.580
Model 5			
HF with EF ≥ 50%	6.79	1.50–30.68	0.013
Creatinine (1 mg/dL increase)	1.24	0.950–01.47	0.092
Model 6			
HF with EF ≥ 50%	7.36	1.51–35.83	0.013
Sodium (1 mEq/L increase)	0.93	0.79–1.12	0.404
Model 7			
HF with EF ≥ 50%	5.31	1.18–23.87	0.030
Recurrence of any atrial tachyarrhythmias	2.15	0.72–6.40	0.170
Model 8			
HF with EF ≥ 50%	6.27	1.37–28.81	0.018
Additional liner ablation	1.11	0.39–3.15	0.841

CI, confidence interval. Other abbreviations are the same as in Table 1.

**Table 4:** All cases of the composite endpoints.

Case No.	Age at Enrollment	Gender	Underlying Heart Disease	Type of AF	CHA2DS2-VASc	HAS-BLED	Outcomes
HF with impaired EF							
1	79	Female	None	Long-persistent	4	1	HF with AF recurrence
2	70	Male	Hypertrophic cardiomyopathy	Paroxysmal	4	1	HF without AF recurrence
HF with preserved EF							
1	75	Male	None	Paroxysmal	4	2	Stroke
2	79	Female	None	Paroxysmal	5	1	Gastrointestinal bleeding
3	64	Male	Ischemia	Persistent	2	2	HF with AF recurrence
4	67	Male	Hypertrophic cardiomyopathy	Paroxysmal	3	2	HF without AF recurrence
5	84	Female	None	Paroxysmal	4	1	HF due to gastrointestinal bleeding
6	86	Female	None	Paroxysmal	4	1	HF due to gastrointestinal bleeding
7	78	Male	None	Long and persistent	3	1	Stroke
8	86	Male	None	Paroxysmal	5	1	HF with AF recurrence
9	76	Female	Amyloidosis	Paroxysmal	5	3	HF with AF recurrence
10	83	Male	None	Paroxysmal	3	1	Sudden cardiac death
11	79	Female	None	Paroxysmal	5	1	HF with AF recurrence
12	68	Male	None	Paroxysmal	5	4	Bleeding from the puncture site
13	74	Male	None	Paroxysmal	6	3	HF with AF recurrence

Abbreviations are the same as in Table 1.

## Data Availability

Derived data supporting the findings of this study are available from the corresponding author on request.
